# Biocontrol Potential of *Trichoderma* spp. Against Fungal Pathogens Associated with Fruit Diseases

**DOI:** 10.3390/jof12100757

**Published:** 2026-10-09

**Authors:** Francisca Pereira, Inês Mendonça, Diana Sousa, Miguel Leão de Sousa, Lúcia Noronha, Armando Venâncio, Sónia Silva

**Affiliations:** 1 CEB—Centre of Biological Engineering, University of Minho, Campus de Gualtar, 4710-057 Braga, Portugal; pg56228@alunos.uminho.pt (F.P.); id11320@alunos.uminho.pt (I.M.); dianasousa14@hotmail.com (D.S.); soniasilva@ceb.uminho.pt (S.S.); 2 INIAV—National Institute for Agrarian and Veterinary Research, I.P., 4485-655 Vairão, Portugal; 3 Colab4Food—Collaborative Laboratory for Innovation in the Agri-Food Industry, 4485-655 Vairão, Portugal; lucia.noronha@colab4food.com; 4 INIAV—National Institute for Agrarian and Veterinary Research, I.P., 2460-059 Alcobaça, Portugal; 5 LABBELS—Associate Laboratory, 4710-057 Braga, Portugal

**Keywords:** antagonistic activity, biological control agents, fruit fungal diseases, low-residue strategies, sustainable agriculture, *Trichoderma* spp.

## Abstract

Fungal contamination is a major constraint in fruit production and postharvest management, highlighting the need for sustainable alternatives to conventional chemical fungicides. This study evaluated the antagonistic potential and biocontrol mechanisms of different *Trichoderma* spp. against four fruit-associated fungal pathogens, namely *Talaromyces trachyspermus*, *Paecilomyces variotii*, *Stemphylium vesicarium*, and *Alternaria arborescens*. The antagonistic activity of *Trichoderma* strains was assessed using dual-culture assays, while the antifungal activity of culture supernatants and the morphological interactions between *Trichoderma* and the target pathogens were examined to elucidate possible mechanisms of antagonism. All tested *Trichoderma* strains exhibited inhibitory activity against the target pathogens, with inhibition rates ranging from 32% to 100%. Among the tested strains, *Trichoderma viride* and *Trichoderma harzianum* showed the strongest overall antagonistic activity. In addition, culture supernatants of *Trichoderma atroviride* and *Trichoderma longibrachiatum* retained antifungal activity, indicating that extracellular metabolites may contribute to pathogen inhibition. Microscopic observations further revealed several mycoparasitic interactions, including adhesion to pathogen hyphae, hyphal coiling, penetration, morphological deformation, and hyphal lysis. Overall, the results demonstrate that the antagonistic activity of *Trichoderma* is both strain- and pathogen-specific and involves multiple complementary mechanisms. These findings highlight the potential of selected *Trichoderma* strains as biological control agents for managing fruit-associated fungal pathogens and as sustainable alternatives to chemical fungicides in fruit production and postharvest disease management.

## 1. Introduction

Maintaining fruit quality and safety remains a key challenge throughout the fruit supply chain, particularly as consumers demand fresh products with high nutritional value, appealing sensory characteristics and recognized health benefits [1,2]. In recent years, consumers demand for high-quality fresh fruit has increased, reinforcing the need to ensure products with high commercial and microbiological standards [3]. However, fruits are perishable products [4], and their composition, particularly their high-water content and acidic pH, creates favorable conditions for fungal disease development [5].

Along the fruit supply chain, fungal contamination may occur from field production to postharvest handling, processing and consumption [6]. This continuous exposure to biological, environmental and handling-related sources of microorganisms makes their complete exclusion from fruit surfaces particularly difficult [7]. Consequently, fungal spoilage represents a persistent problem for both fresh fruit and fruit-derived products, with implications for food quality, safety and economic losses [8].

Among the fungal diseases affecting fruit production, brown spot of pear (BSP) is particularly important in European pear-producing regions. Caused by *Stemphylium vesicarium* (Wall.) E. Simmons, this disease has been associated with considerable economic losses [9]. BSP symptoms may develop on fruits, leaves and shoots, typically as necrotic lesions. As the disease progresses, lesions on fruit tend to expand and may facilitate secondary colonization by saprophytic fungi, namely *Alternaria arborescens*, thereby contributing to fruit rotting and loss of commercial value [10].

Fungal spoilage is also a major concern in processed fruit products and may be associated with the fruit raw materials used for their production [11]. *Paecilomyces variotii* is considered an important spoilage fungus due to its rapid growth, thermotolerance, ability to grow under low-oxygen conditions, tolerance to preservatives, and capacity to produce the mycotoxin viriditoxin [12,13]. *Talaromyces* spp., particularly *Talaromyces trachyspermus*, are also among the most frequently reported fungi in processed fruit products [13], making these fungi highly relevant to the beverage industry [14].

The management of fruit fungal diseases has traditionally relied on chemical treatments, particularly fungicides, which remain among the most widely used strategies for fungal control [15]. Together with resistant crops and appropriate management practices, chemical pesticides have contributed to increased crop yield and improved product quality over recent decades. Nevertheless, their intensive and repeated application has raised important concerns. Excessive pesticide use has been associated with environmental pollution, which restricts their sustainable application in agriculture [16,17]. In addition, fungicide use has been increasingly questioned due to ecotoxicity, potential risks to human health, high costs and the emergence of resistance in phytopathogenic microorganisms [18].

These limitations have strengthened the search for alternative strategies that are safer for consumers and less harmful to the environment [19]. In this context, biological control agents (BCAs) have gained attention as a promising approach for managing fungal diseases in fruit systems. Biocontrol may be understood as the use of naturally occurring microorganisms, controlled microflora and/or their antimicrobial products to improve the safety of fresh produce and extend its shelf life [20].

Among the different fungal antagonists explored for the control of fungal diseases, *Trichoderma* has been highlighted as one of the genera with the greatest biocontrol potential [21].

*Trichoderma* comprises a genus of filamentous fungi that is generally described as saprophytic, avirulent and opportunistic plant symbionts, mainly associated with soil environments [22]. The biocontrol activity of *Trichoderma* spp. is supported by multiple mechanisms. Some of these act directly against pathogens, including mycoparasitism, competition for space and nutrients, antibiosis through the production of antimicrobial compounds, and secretion of lytic enzymes. Other mechanisms act indirectly by strengthening the plant, namely through the induction of systemic resistance, the promotion of plant growth and rhizosphere competence [23]. This diversity of mechanisms, together with their capacity to adapt to different environments, has supported the use of *Trichoderma* spp. in the control of fungal plant diseases. In addition, these fungi may provide further benefits to agriculture, such as improved photosynthetic capacity, increased yields, enhanced nutrient absorption and greater tolerance to abiotic stress [24].

In light of the importance of fruit-associated fungal pathogens throughout the fruit supply chain, as well as the need for safer and more sustainable control strategies, this study aims to evaluate the biocontrol potential of *Trichoderma* spp. against fungal pathogens associated with fruit production and processing.

Considering the occurrence of fungal contamination at different stages of the fruit supply chain and the increasing interest in sustainable disease management strategies, this study aimed to evaluate the in vitro biocontrol potential of selected *Trichoderma* spp. against fungal pathogens associated with fruit production and processing. The novelty of this study lies in the comparative evaluation of multiple *Trichoderma* strains against a diverse set of fungal targets differing in their biological characteristics, ecological niches and relevance within the fruit supply chain, rather than restricting the analysis to a single *Trichoderma* spp./strains or a single pathogen–antagonist interaction. Specifically, their antagonistic activity was assessed against *S. vesicarium* and *A. arborescens*, associated with pear production, as well as *P. variotii* and *T. trachyspermus*, relevant spoilage fungi in fruit-derived products. The study therefore provides a comparative assessment of the antagonistic performance of *Trichoderma* against fungal targets representing distinct stages of the fruit supply chain, contributing to the identification of promising strains for subsequent validation under more complex biological conditions.

## 2. Materials and Methods

### 2.1. Microorganisms

#### 2.1.1. Fruit-Associated Fungal Pathogens

Seven fungal strains were used in this study as target pathogens (Table 1). All isolates were originally isolated and identified by members of the Fungi Research Group of University of Minho (UM) from infected fruit material or contaminated fruit-derived products and preserved in Micoteca da Universidade do Minho culture collection (MUM, Braga, Portugal). The selected strains included phytopathogenic fungi associated with ‘Rocha’ pear BSP disease, namely *S. vesicarium* and *A. arborescens*, as well as spoilage fungi, including *T. trachyspermus* and *P. variotii*, isolated from fruit juice matrices. The fungal strains were preserved at room temperature in vials containing sterile distilled water. For experimental use, the strains were grown on Potato Dextrose Agar (PDA; Liofilchem, Roseto degli Abruzzi, Italy) at 25 °C for 6 days and subsequently stored at 4 °C until further use.

The identification of the fungal pathogen isolates was molecularly confirmed by Sanger sequencing following the methodology described by Mendonça et al. [25]. The internal transcribed spacer (ITS) region was amplified using primers ITS1 (5′-TCCGTAGGTGAACCTGCGG-3′) and ITS4 (5′-TCCTCCGCTTATTGATATGC-3′), while the glyceraldehyde-3-phosphate dehydrogenase (gpd) gene was amplified using primers Gpd1 (5′-CAACGGCTTCGGTCGCATTG-3′) and Gpd2 (5′-GCCAAGCAGTTGGTTGTGC-3′).

#### 2.1.2. *Trichoderma* Strains

*Trichoderma* strains (n = 10) were obtained from MUM. The species and corresponding strain codes are presented in Table 2.

All *Trichoderma* strains listed in Table 2 were grown on PDA and incubated at 25 °C for 6 days. The cultures were then stored at 4 °C until further use. *Trichoderma* strains were initially identified by matrix-assisted laser desorption/ionization time-of-flight mass spectrometry (MALDI-TOF MS) (Bruker, Bremen, Germany) and their identification was subsequently confirmed by molecular analysis. For this purpose, a fragment of approximately 400 bp of the translation elongation factor 1-alpha (TEF1-α) gene was amplified using the primer pair EF1-728F (5′-CATCGAGAAGTTCGAGAAGG-3′) and EF1-986R (5′-TACTTGAAGGAACCCTTACC-3′), described by Carbone and Kohn (1999), followed by Sanger sequencing [26].

### 2.2. Fruit-Associated Fungal Pathogens Sensitivity to Chemical Fungicides

To assess fruit-associated fungal pathogen sensitivity to commercial fungicides, six fungicides—Metalaxyl-M + Cymoxanil, Kresoxim-methyl + Difenoconazole, Difenoconazole + Mandipropamid, Azoxystrobin + Difenoconazole, Cyprodinil + Fludioxonil, and Cyflufenamid + Difenoconazole—were evaluated using the poisoned food technique [27]. For this purpose, PDA was supplemented with the respective fungicide at the recommended field concentrations application (Table 3). Control plates consisted of PDA without fungicide addition. After solidification, each plate was inoculated at the center with a 5 mm × 5 mm mycelial plug of the respective fungi pathogen and incubated at 25 °C for 10 days. During the incubation period, colony diameter was measured, and fungal growth inhibition factor was calculated according to Equation (1).

(1) $$\mathrm{Growth}\;\mathrm{inhibition}\;\mathrm{factor}\;(\%)=\frac{A-B}{A}\times 100,$$

where:

A: the diameter of the control colony without treatment (cm); B: the diameter of the colony with treatment (cm).

**Table 3:** Chemical fungicides tested against fruit-associated fungal pathogens.

Active Ingredient	Group Name	FRAC ^i^ Group Code	FRAC Level of Resistance Risk	Concentration Tested
4% (p/p) Metalaxyl-M + 4.8% (p/p) Cymoxanil	Phenylamides	4	High	30 g/hL
	Cyanoacetamideoxime	27	Low to Medium	
25% (p/p) Kresoxim-methyl + 12.5 (p/p) Difenoconazole	Quinone outside inhibitors	11	High	30 g/hL
	Demethylation inhibitions	3	Medium	
21.8% (p/p) Difenoconazole + 21.8% (p/p) Mandipropamid	Demethylation inhibitions	3	Medium	60 mL/hL
	Carboxylic acid amides	40	Low to Medium	
18% (p/p) Azoxystrobin + 11.3% (p/p) Difenoconazole	Quinone outside inhibitors	11	High	100 mL/hL
	Demethylation inhibitions	3	Medium	
37.5% (p/p) Cyprodinil + 25% (p/p) Fludioxonil	Anilinopyrimidines	9	Medium	100 g/hL
	Phenylpyrroles	12	Low to Medium	
2.8% (p/p) Cyflufenamid + 5.6% (p/p) Difenoconazole	Phenyl-acetamide	U 06	*	65 mL/hL
	Demethylation inhibitions	3	Medium	

^i^ FRAC—fungicide resistance action committee. * Resistance in Sphaerotheca Resistance Management required [28].

### 2.3. Determination of the Growth Rates of Fruit-Associated Fungal Pathogens and Trichoderma

The growth performance of the fruit-associated fungal pathogens and the *Trichoderma* strains was assessed on PDA at 25 °C for 13 days (corresponding to the finite agar surface). For that, mycelial plugs collected from the actively grown margin of 6-day old cultures of all fungi were used. Fragments measuring 5 mm × 5 mm were transferred to the center of 9 cm Petri plates with PDA. The colony diameter was measured every day and for each measurement, the initial inoculum size was subtracted from the observed colony diameter.

Growth parameters were calculated by fitting the Baranyi and Roberts model [29] to the colony diameter data. The model was used to estimate two growth parameters: the maximum mycelial growth rate, μ (cm h^−1^), and the lag phase, λ (h).

### 2.4. Screening of Antagonistic Activity of Trichoderma Against Fruit-Associated Fungal Pathogens

#### Dual-Culture Assay

The antagonistic activity of *Trichoderma* strains against the fruit-associated fungal pathogens was evaluated using the dual-culture technique [30]. The fungal antagonists and fungal pathogens were inoculated onto freshly prepared PDA. Each *Trichoderma* was inoculated on one side of the plate, approximately 1 cm from the edge, while each fungal pathogen was inoculated on the opposite side of the same plate, also approximately 1 cm from the edge. Control plates were prepared under the same conditions; however, instead of pairing a *Trichoderma* with a fungal pathogen, the same fungal pathogen was inoculated on both opposite sides of the PDA. All plates were incubated at 25 °C for 10 days.

After incubation, the radial growth of the fungal pathogen in the control and dual-culture plates was measured. The percentage inhibition of radial growth (PIRG) was then calculated according to Equation (2).

(2) $$\mathrm{PIRG}\;(\%)=\frac{A-B}{A}\times 100,$$

where:

A: the radial growth of the fungal pathogen in the control plates (cm); B: the radial growth of the phytopathogen in the presence of *Trichoderma* (cm).

### 2.5. Antifungal Activity of Cell- and Spore-Free Culture Supernatant of Trichoderma Against Fruit-Associated Fungal Pathogens

#### 2.5.1. Production of Cell- and Spore-Free Culture Supernatants from *Trichoderma*

For the evaluation of the antifungal activity of *Trichoderma* cell- and spore-free culture supernatants, the five *Trichoderma* strains that exhibited the highest antagonistic activity in the dual-culture assays (MUM 02.21, MUM 14.18, MUM 22.23, MUM 22.25, and MUM 23.09) were selected.

For that, each *Trichoderma* strain was grown in 100 mL of Potato Dextrose Broth (PDB, Liofilchem, Roseto degli Abruzzi, Italy) and incubated for 7 days at 25 °C at 150 rpm. The cultures were subjected to vacuum filtration using Fisherbrand qualitative filter paper to separate the fungal biomass from the liquid culture fraction. The retained biomass was removed, and the resulting liquid filtrate was used in the subsequent steps. To obtain cell- and spore-free culture supernatants, the liquid fraction was further sterilized by filtration using FilterBio sterile syringe filters with a 0.22 µm membrane pore. The pH of each fungal liquid culture was determined using an edge^®^ pH meter (Hanna Instruments, Woonsocket, RI, USA). All PDB media had an initial pH of 5.6 prior to fungal inoculation. The obtained culture supernatants were stored at 4 °C until their use in antifungal activity assays.

#### 2.5.2. Antifungal Activity of *Trichoderma* Culture Supernatants

The antifungal activity of the obtained *Trichoderma* cell- and spore-free culture supernatants was assessed by incorporating them into previously autoclaved PDA medium. For this purpose, PDA was prepared using 25% less water than normally required, and the missing volume was subsequently replaced with the culture supernatant, resulting in a final supernatant concentration of 25% (*v*/*v*) [31]. Each plate was then inoculated with a 5 mm × 5 mm mycelial plug of each fungal pathogen. Control plates consisted of non-supplemented PDA inoculated under the same conditions. All plates were incubated at 25 °C for 10 days. The radial growth diameter of each fungal pathogen was measured, and the percentage of mycelial growth inhibition (MGI) was calculated according to Equation (2).

#### 2.5.3. Effect of Autoclaving *Trichoderma* Culture Supernatants

Based on the results obtained for antifungal activity of *Trichoderma* culture supernatants against fungal pathogens, *T. longibrachiatum* (MUM 14.18) and *T. atroviride* (MUM 02.21) were selected for autoclaving assay as the two best-performing strains. Briefly, culture supernatants obtained from liquid cultures of the selected *Trichoderma* strains were autoclaved at 121 °C for 15 min. After cooling, the autoclaved supernatants were incorporated into PDA and the fungal inoculated as described in Section 2.5.2. Control consisted of PDA supplemented with non-autoclaved culture filtrate.

After incubation, the mycelial growth of each fruit-associated fungal pathogens was measured in PDA supplemented with autoclaved and non-autoclaved culture filtrate. The results were expressed as a percentage of reduction in the antifungal *Trichoderma* strains compared to the non-autoclaved supernatants.

### 2.6. Mycoparasitism Relationship Among Trichoderma and Fruit-Associated Fungal Pathogens

#### Slide Culture Technique

The slide culture technique described by Santos et al. [32] was used to analyze the microscopic interactions between *Trichoderma* strains and the fungal pathogens. For that, a humid chamber was prepared using a sterile 90 mm Petri dish containing sterile paper moistened with approximately 1 mL of sterile distilled water. Two sterile wooden supports were placed in parallel over the moistened paper, and a sterile glass slide was positioned on top to avoid direct contact with the wet surface.

A small agar block, approximately 7 mm wide and 5 mm thick, was aseptically cut from solidified PDA using a sterile scalpel and placed in the center of the glass slide. The agar block was inoculated with *Trichoderma* on one side and the corresponding fungal pathogen on the opposite side. The agar blocks were carefully covered with a sterile coverslip. The Petri plates were sealed and incubated at 25 °C for 5 days.

After incubation, the coverslip was gently removed from the agar block to minimize disruption of the fungal structures. The prepared slides were observed using an Olympus BX51 optical microscope (Olympus, Tokyo, Japan) under bright-field and dark-field conditions to evaluate hyphal growth, and the interaction patterns between *Trichoderma* strains and the fungal pathogens. Images were observed using a 20× objective.

### 2.7. Statistical Analysis

Data were statistically analyzed using GraphPad Prism 11.0 (GraphPad Software, Inc., La Jolla, CA, USA). Experiments were performed at least in triplicate, and data are presented as mean ± standard error of the mean. For datasets involving the simultaneous comparison of *Trichoderma* strains and fungal pathogens, an ordinary two-way analysis of variance (ANOVA) was performed to assess the effects of both factors and their interaction, followed by Tukey’s multiple comparisons test. When multiple groups were compared within a single factor, an ordinary one-way ANOVA followed by Tukey’s multiple comparisons test was used. Comparisons between two independent groups were performed using an unpaired *t*-test with Welch’s correction. Differences were considered statistically significant at *p* < 0.05.

## 3. Results

### 3.1. Fruit-Associated Fungal Pathogens Sensitivity to Chemical Fungicides

**Figure 1:**
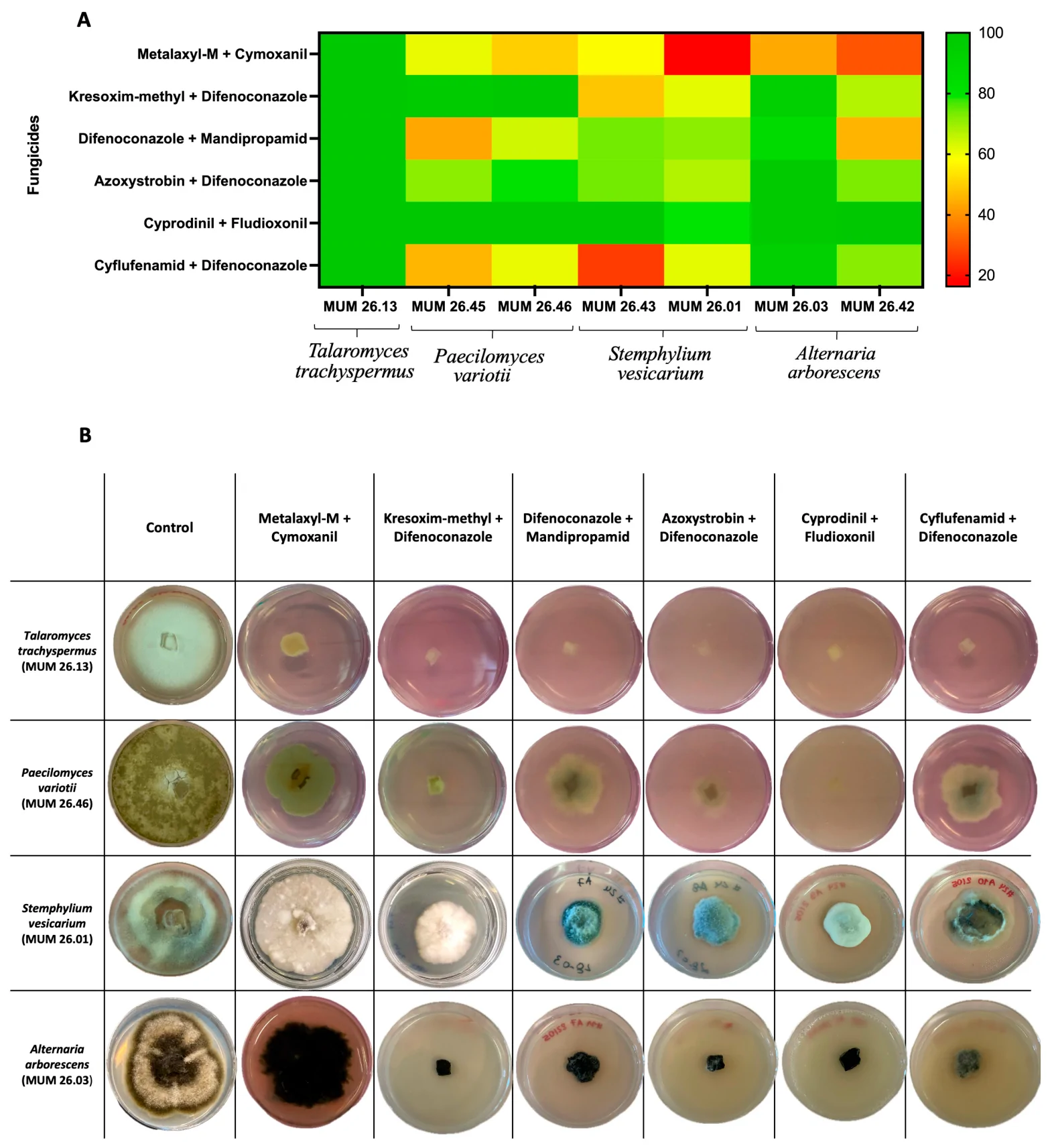
Fruit-associated fungal pathogens sensitivity to chemical fungicides cultured on PDA at 25 °C. (**A**) A heat map showing the inhibition factor (%) of fungal pathogens following exposure to chemical fungicides. Green indicates higher growth inhibition and red indicates lower inhibition. (**B**) Representative images of fungal pathogens grown on PDA in the absence and presence of the fungicide.

The sensitivity of the fungal pathogen isolates to six commercial fungicides was evaluated and the growth inhibition factors obtained for each fungicide-isolate combination are summarized in Figure 1A. Overall, the inhibition response varied markedly according to both the fungicide formulation and the fungal isolate tested.

Cyprodinil + Fludioxonil was the most effective fungicide under the tested conditions, followed by Kresoxim-methyl + Difenoconazole and Azoxystrobin + Difenoconazole. In contrast, Metalaxyl-M + Cymoxanil and Cyflufenamid + Difenoconazole showed the most isolate-dependent activity, indicating differences in fungicide susceptibility among the fungi.

**Figure 2:**
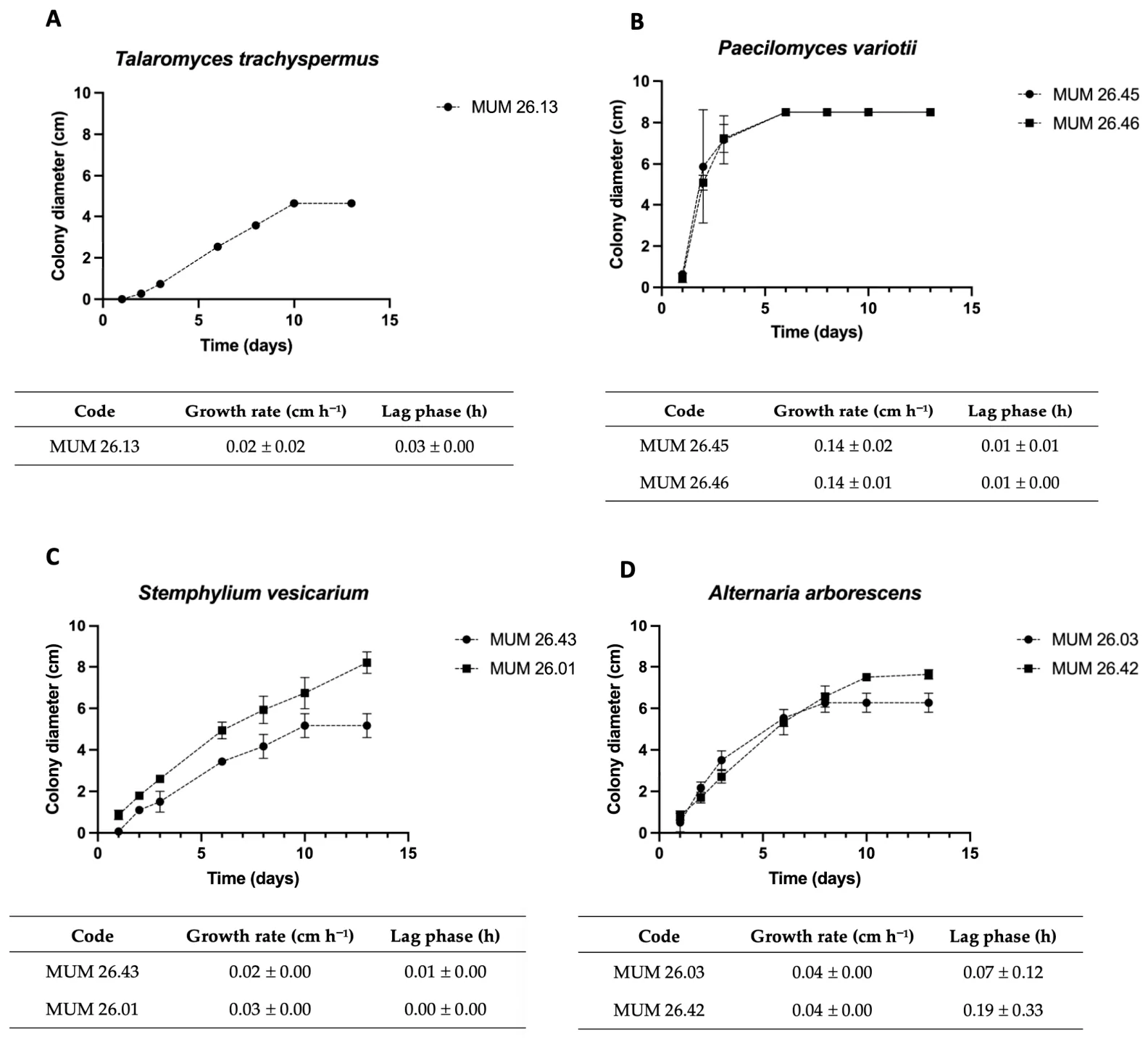
Growth curves and estimated growth parameters of fruit-associated fungal pathogens. (**A**) *S. vesicarium*, (**B**) *A. arborescens*, (**C**) *P. variotii* and (**D**) *T. trachyspermus* cultured on PDA at 25 °C.

Regarding the fungal pathogens, *T. trachyspermus* exhibited the highest susceptibility to all fungicides tested. In contrast, *P. variotii* strains showed the lowest susceptibility to Cyflufenamid + Difenoconazole, Difenoconazole + Mandipropamid, and Metalaxyl-M + Cymoxanil. For *S. vesicarium*, the susceptibility profile varied between isolates. *S. vesicarium* (MUM 26.43) was less affected by Cyflufenamid + Difenoconazole, whereas *S. vesicarium* (MUM 26.01) exhibited a weaker response to Metalaxyl-M + Cymoxanil. Between the *A. arborescens* strains (MUM 26.03) showed reduce sensitivity to Metalaxyl-M + Cymoxanil, while MUM 26.42 exhibited lower susceptibility to both Metalaxyl-M + Cymoxanil and Difenoconazole + Mandipropamid. Figure 2B shows representative images supporting the quantitative results presented in Figure 2A.

### 3.2. Growth Rate of Fruit-Associated Fungal Pathogens and Trichoderma

The growth kinetics of the seven fungal pathogens strains were evaluated on PDA at 25 °C. Colony diameter progression over the incubation period is shown in Figure 2, together with the growth parameters, including the maximum mycelial growth rate (μ) and lag phase (λ).

Regarding growth rate, the fastest-growing isolates were *P. variotii* (MUM 26.45 and MUM 26.46) with growth rates considerably higher than those observed for the remaining fungal pathogens. In contrast, *T. trachyspermus* and *S. vesicarium* (MUM 26.43) showed the lowest growth rates, both with 0.02 cm h^−1^. The longest lag phase was observed for *A. arborescens* (MUM 26.03) and *T. trachyspermus*, whereas the two *S. vesicarium* and *P. variotii* strains exhibited the shortest lag phases under the tested conditions.

**Figure 3:**
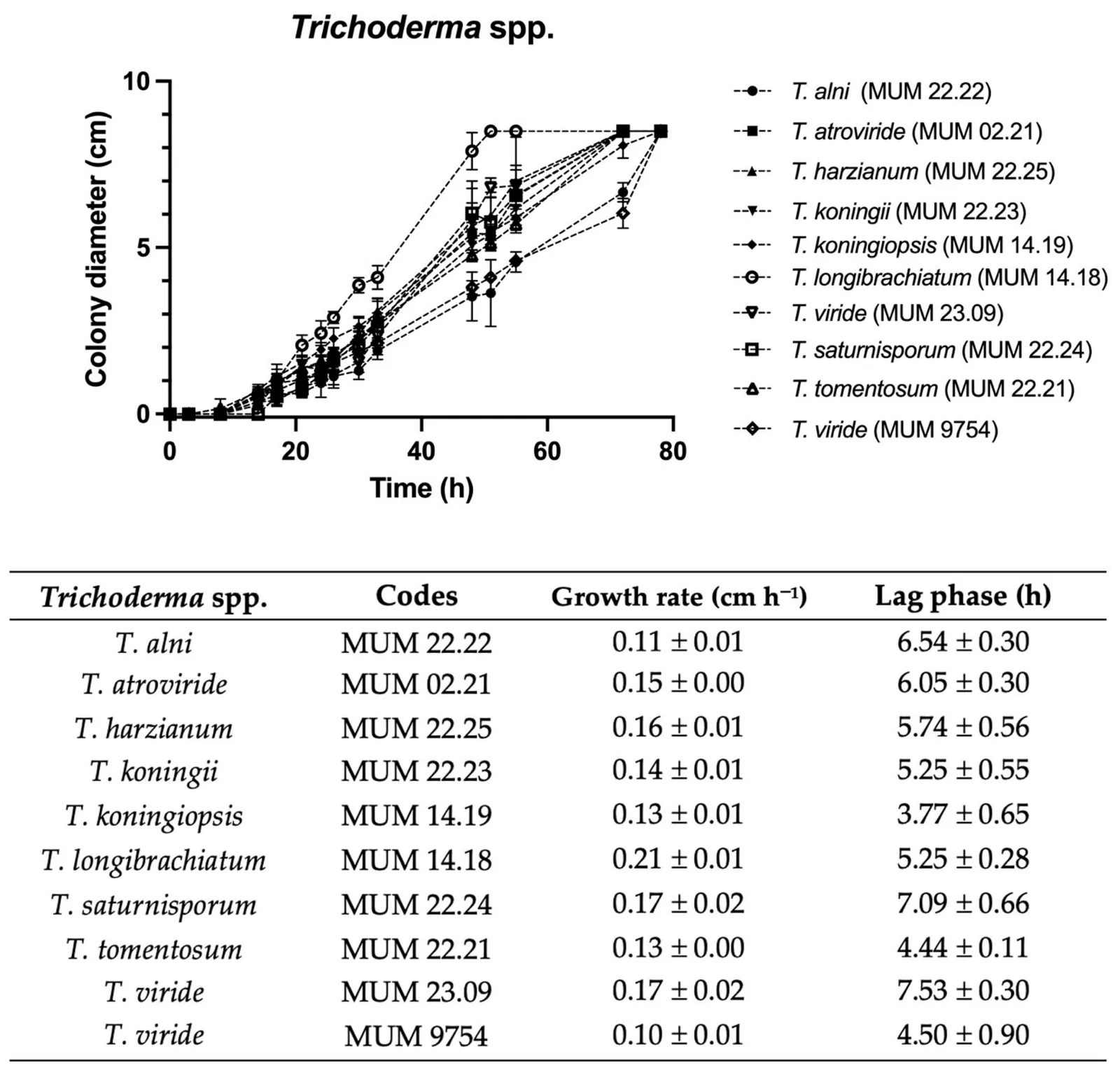
Growth curves and estimated growth parameters of *Trichoderma* strains cultured on PDA at 25 °C.

The growth rates of the ten *Trichoderma* strains were also evaluated in same conditions and are presented in Figure 3. Differences in growth rate were observed among the strains, with *T. longibrachiatum* (MUM 14.18) showing the highest value (0.21 ± 0.01 cm h^−1^), while the lowest was recorded for *T. viride* (MUM 9754; 0.10 ± 0.01 cm h^−1^). Variations were also observed in the estimated lag phase, ranging from 3.77 ± 0.65 h for *T. koningiopsis* (MUM 22.23) to 7.53 ± 0.30 h for *T. viride* (MUM 23.09).

### 3.3. Screening of Antagonistic Activity of Trichoderma Against Fruit-Associated Fungal Pathogens

#### Antagonism Dual-Culture Assays

**Figure 4:**
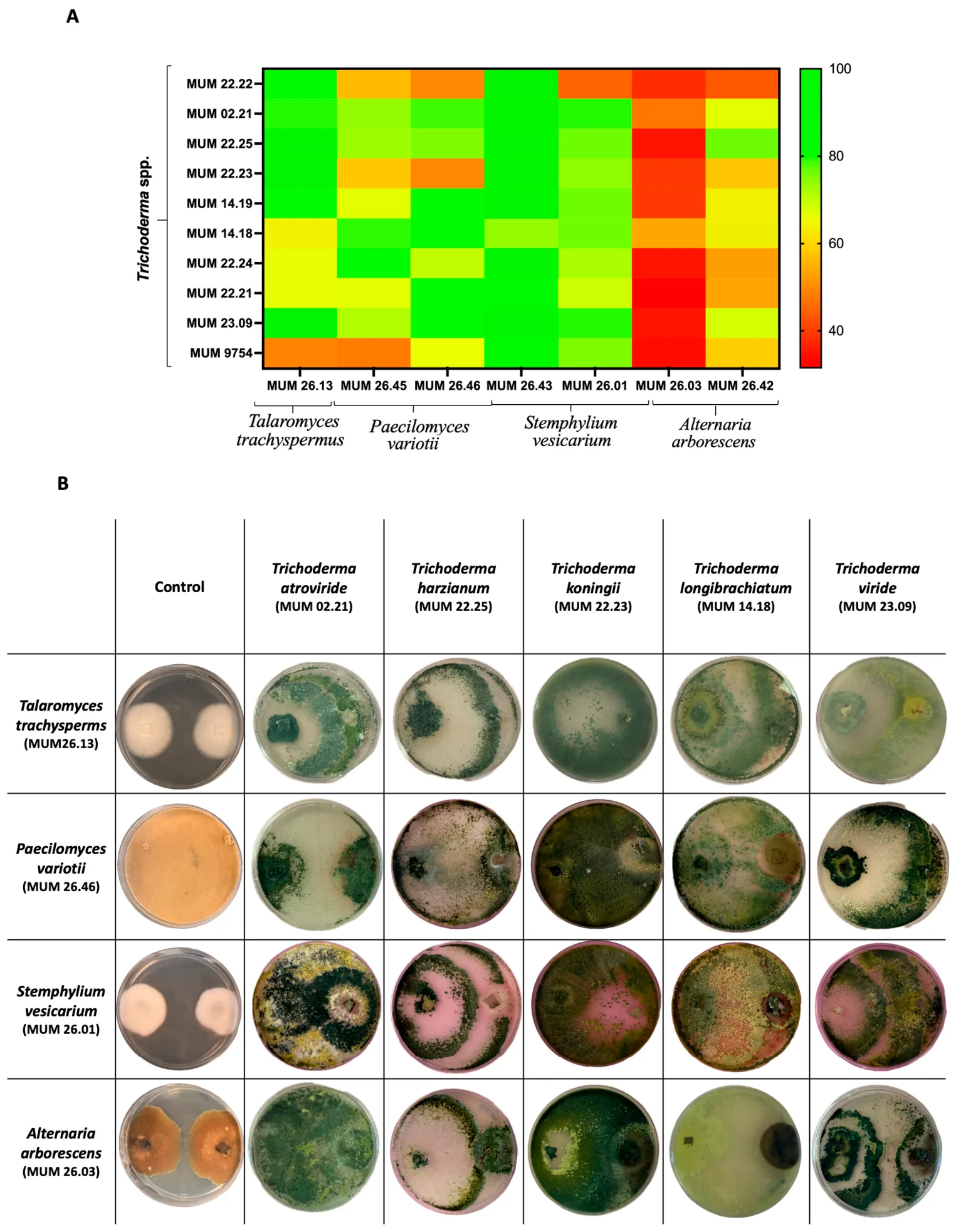
The antagonistic activity of *Trichoderma* strains against fruit-associated fungal pathogens evaluated by dual culture assay on PDA at 25 °C. (**A**) A heat map showing the inhibition factor (%) of fungal pathogens. Green indicates higher growth inhibition and red indicates lower growth inhibition. (**B**) Representative images of dual-culture assays illustrating the interactions between *Trichoderma* strains and the target fungal pathogens. *Trichoderma* spp. are positioned on the left and the target fungus on the right.

Dual-culture assays showed that all *Trichoderma* strains inhibited the growth of fungal pathogens, although the degree of inhibition varied depending on both the *Trichoderma* strain and the target fungal pathogen (Figure 4).

Overall, growth inhibition values ranged from 32% to 100%, indicating a clear isolate-dependent antagonistic response to the fungal pathogen.

Among the tested antagonists, *T. viride* (MUM 23.09) and *T. harzianum* (MUM 22.25), exhibited the highest overall inhibition, with mean inhibition percentages of 77%, followed by *T. atroviride* (MUM 02.21, 75%). Intermediate inhibition levels were observed for *T. koningii* (MUM 22.23), *T. saturnisporum* (MUM 22.24), *T. tomentosum* (MUM 22.21), and *T. viride* (MUM 9754), with mean inhibition values ranging from 60% to 70%. The lowest overall inhibition was observed for *T. alni* (MUM 22.22), with a mean inhibition of 59%.

Regarding the fungal pathogens, *T. trachyspermus* was more susceptible to *T. viride* (MUM 23.09), *T. koningii* (MUM 22.23), and *T. harzianum* (MUM 22.25), showing complete growth inhibition (100%). In contrast, *T. viride* (MUM 9754) was the least effective antagonist against this pathogen, with an inhibition of 49%. *P. variotii* strains were less inhibited by *T. viride* (MUM 9754), *T. koningii* (MUM 22.23) and *T. alni* (MUM 22.22), with values of inhibition ranging from 48% to 66%. *S. vesicarium* (MUM 26.43) exhibited the highest inhibition rates among all fungal pathogens tested against most *Trichoderma* strains with inhibition values ranging from 74% to 100%. In contrast, *A. arborescens* (MUM 26.03) exhibited the lowest inhibition rates among all fungal pathogens tested against most *Trichoderma* strains, with values ranging from 32% to 54%.

### 3.4. Antifungal Activity of Cell- and Spore-Free Culture Supernatants of Trichoderma Against Fruit-Associated Fungal Pathogens

Based on the results of the dual-culture assays, five *Trichoderma* strains were selected for further evaluation of the antifungal activity of their cell- and spore-free culture supernatants: *T. atroviride* (MUM 02.21), *T. harzianum* (MUM 22.25), *T. koningii* (MUM 22.23), *T. longibrachiatum* (MUM 14.18), and *T. viride* (MUM 23.09).

#### 3.4.1. pH of *Trichoderma* Culture Supernatants

After 7 days of growth on PDB, the pH of *Trichoderma* supernatants was determined, with the values presented in Table 4.

No marked variability in pH values was observed among the *Trichoderma* culture supernatants, which ranged from approximately 4 to 6. The lowest pH was recorded for *T. koningii* (MUM 22.23), whereas the highest pH value was observed for *T. harzianum* (MUM 22.25).

#### 3.4.2. Antifungal Activity of *Trichoderma* Culture Supernatants

**Figure 5:**
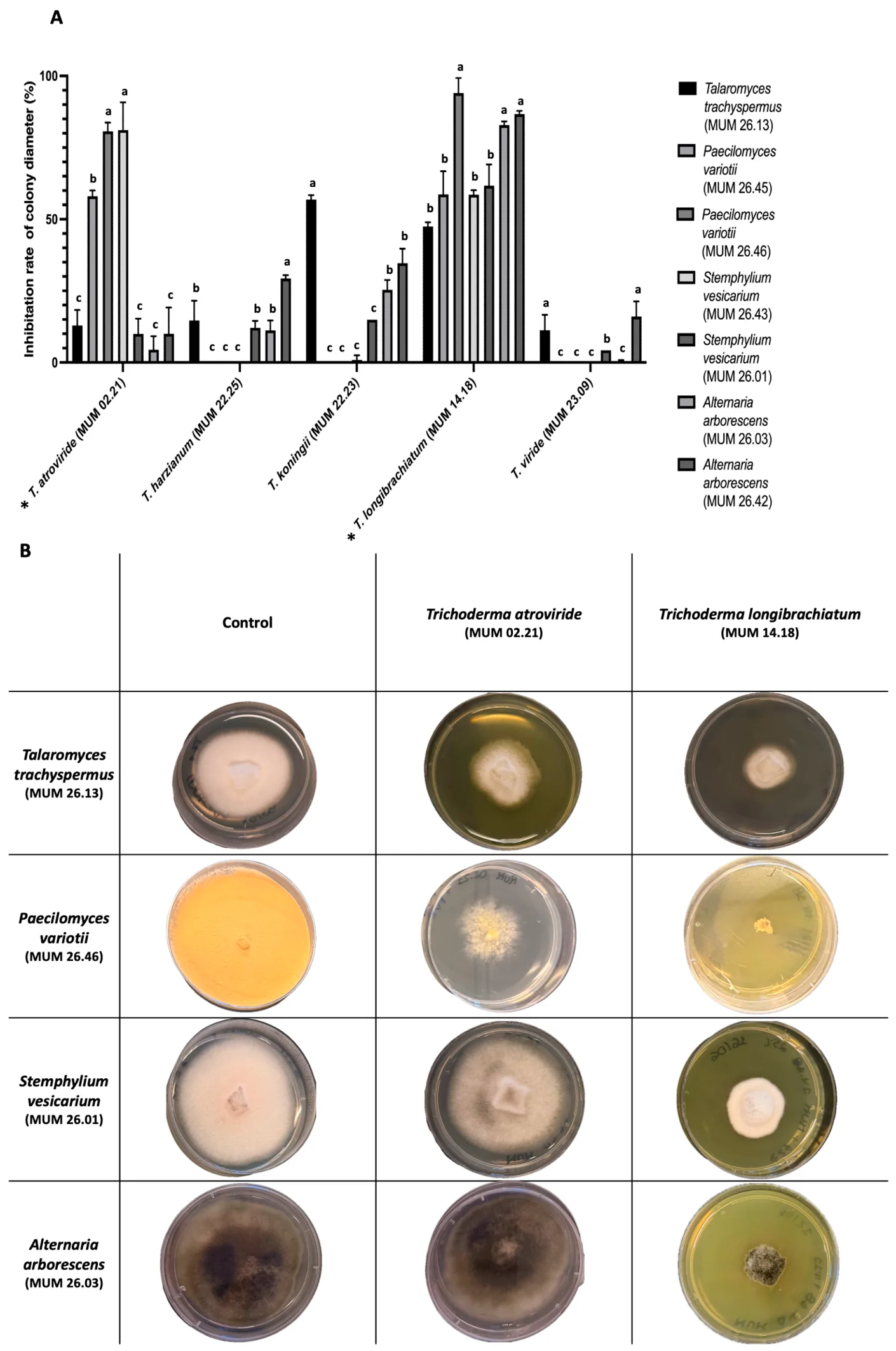
The antifungal activity of *Trichoderma* culture supernatants against fruit-associated fungal pathogens. (**A**) Percentage inhibition of mycelial growth of fungal pathogens by 25% (*v*/*v*) of each *Trichoderma* culture filtrate. Different letters within each *Trichoderma*, indicates statistically significant differences among values obtained for the fungal pathogen (*p* < 0.05). * Indicates a statistically significant difference in inhibitory activity compared with the other *Trichoderma* culture supernatants (*p* < 0.05). (**B**) Representative images demonstrating the inhibitory impact of *Trichoderma* culture supernatants on the growth of the target pathogen.

The antifungal activity of harvested supernatants from *Trichoderma* liquid cultures produced by the five selected *Trichoderma* strains was assessed against the seven fungal pathogens (Figure 5). The inhibitory effect varied according to both the *Trichoderma* strains and the target fungus.

Among the cell- and spore-free culture supernatants tested (Figure 5), those produced by *T. atroviride* (MUM 02.21) and *T. longibrachiatum* (MUM 14.18) exhibited the highest antifungal activity, with significantly greater inhibition compared with the other *Trichoderma* (*p* < 0.05). *T. atroviride* showed particularly high antifungal activity against both *P. variotii* isolates (>75%) and *S. vesicarium* (MUM 26.43; 60%) (*p* < 0.05), whereas considerably lower inhibition was observed against the remaining pathogens. Moreover, *T. longibrachiatum* (MUM 14.18) inhibited the growth of all tested fungal pathogens (Figure 5), with inhibition values exceeding 50%. The highest inhibitory activity, above 90%, was observed against *P. variotii* (MUM 26.46) and both *A. arborescens* isolates (*p* < 0.05; >75%).The culture supernatants produced by *T. harzianum* (MUM 22.25), *T. koningii* (MUM 22.23), and *T. viride* (MUM 23.09) generally showed lower inhibitory activity.

Figure 5B shows representative images supporting the quantitative results showed in Figure 5A.

#### 3.4.3. Effect of Autoclaving *Trichoderma* Culture Supernatants

**Figure 6:**
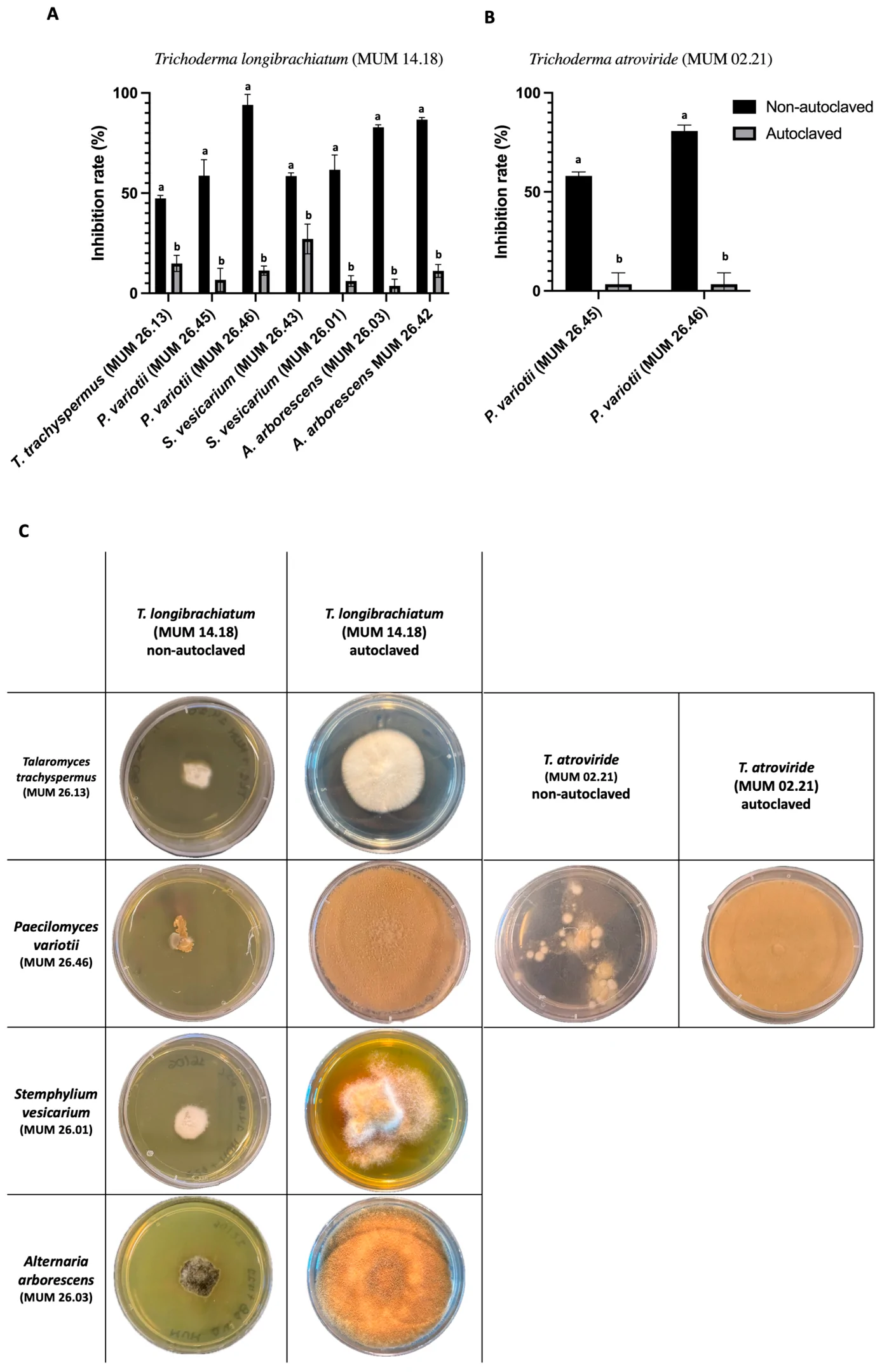
The effect of autoclaving on the antifungal efficacy of *Trichoderma* culture supernatants against fruit-associated fungal pathogens. (**A**) The inhibition rate of fungal pathogens by non-autoclaved and autoclaved culture supernatants of *T. longibrachiatum* (MUM 14.18). (**B**) The inhibition rate of *P. variotii* isolates by non-autoclaved and autoclaved culture supernatants of *T. atroviride* (MUM 02.21). For each pathogen isolate, different letters indicate statistically significant differences between non-autoclaved and autoclaved supernatants (*p* < 0.05). (**C**) Representative images illustrating the effect of autoclaving on the antifungal activity of *Trichoderma* culture supernatants.

Since the culture supernatants of *T. longibrachiatum* (MUM 14.18) and *T. atroviride* (MUM 02.21) exhibited the highest antifungal activity, the supernatants were selected to evaluate the effect of heat treatment on their inhibitory activity by autoclaving. The results are shown in Figure 6 and expressed as the reduction in antifungal activity relative to the non-autoclaved filtrates.

Autoclaving reduced the antifungal activity of the *T. longibrachiatum* (MUM 14.18) culture filtrate against all tested fungal isolates, although the magnitude of this reduction differed significantly among isolates (*p* < 0.05) (Figure 6A). The highest loss of antifungal activity was observed against *P. variotii* (MUM 26.46) and both *A. arborescens* isolates reaching around 70% of reduction. In contrast, the antifungal activity was less affected by autoclaving against *T. trachyspermus* (MUM 26.23) and *S. vesicarium* (MUM 26.43) (*p* < 0.05). Concerning the effect on *P. variotii* (MUM 26.46) and *S. vesicarium* (MUM 26.01) the decrease was approximately 50% (*p* < 0.05). The *T. atroviride* (MUM 02.21) culture filtrate showed a greater reduction in antifungal activity against both *P. variotii* strains, with a more pronounced effect on *P. variotii* (MUM 26.46) (*p* < 0.05). Figure 6C shows representative images supporting the quantitative results showed in Figure 6A,B.

### 3.5. Mycoparasitism Relationship Among Trichoderma and Fruit-Associated Fungal Pathogens

**Figure 7:**
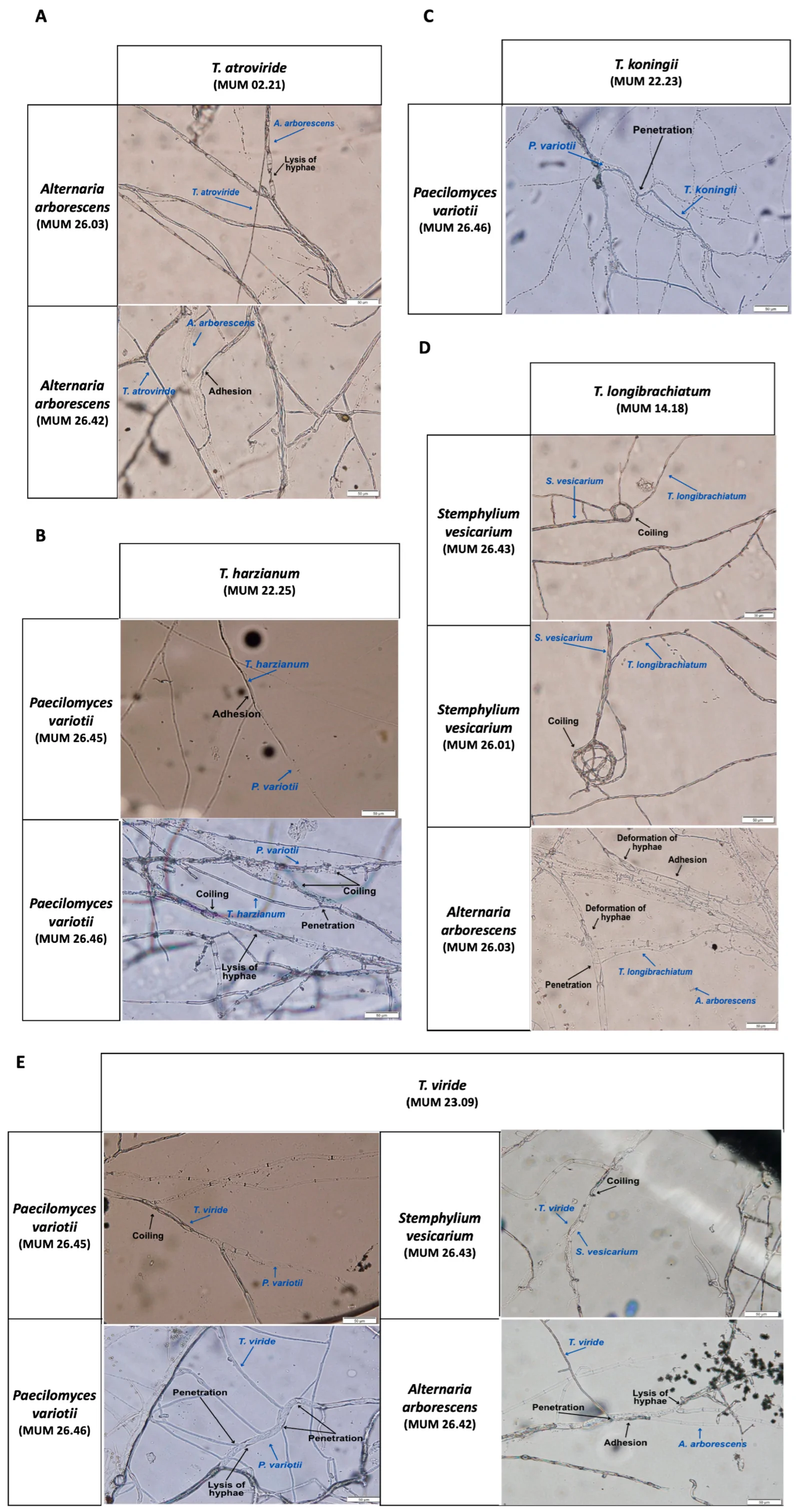
The mycoparasitism relationship among *Trichoderma* and fruit-associated fungal pathogens. Representative hyphal interactions observed between (**A**) *T. atroviride* (MUM 02.21) and *A. arborescens* (MUM 26.03 and MUM 26.42); (**B**) *T. harzianum* (MUM 22.25) and *P. variotii* (MUM 26.46); (**C**) *T. koningii* (MUM 22.23) and *P. variotii* (MUM 26.46); (**D**) *T. longibrachiatum* (MUM 14.18) and *S. vesicarium* (MUM 226.43) and *A. arborescens* (MUM 26.01); (**E**) *T. viride* (MUM 23.09) and *P. variotii* (MUM 26.45) and *S. vesicarium* (MUM 26.43). Representative microscopic images of the corresponding *Trichoderma* strains and target fungi grown individually under the same conditions are shown in Figure A1. Scale bar correspond to 50 μm.

**Figure A1:**
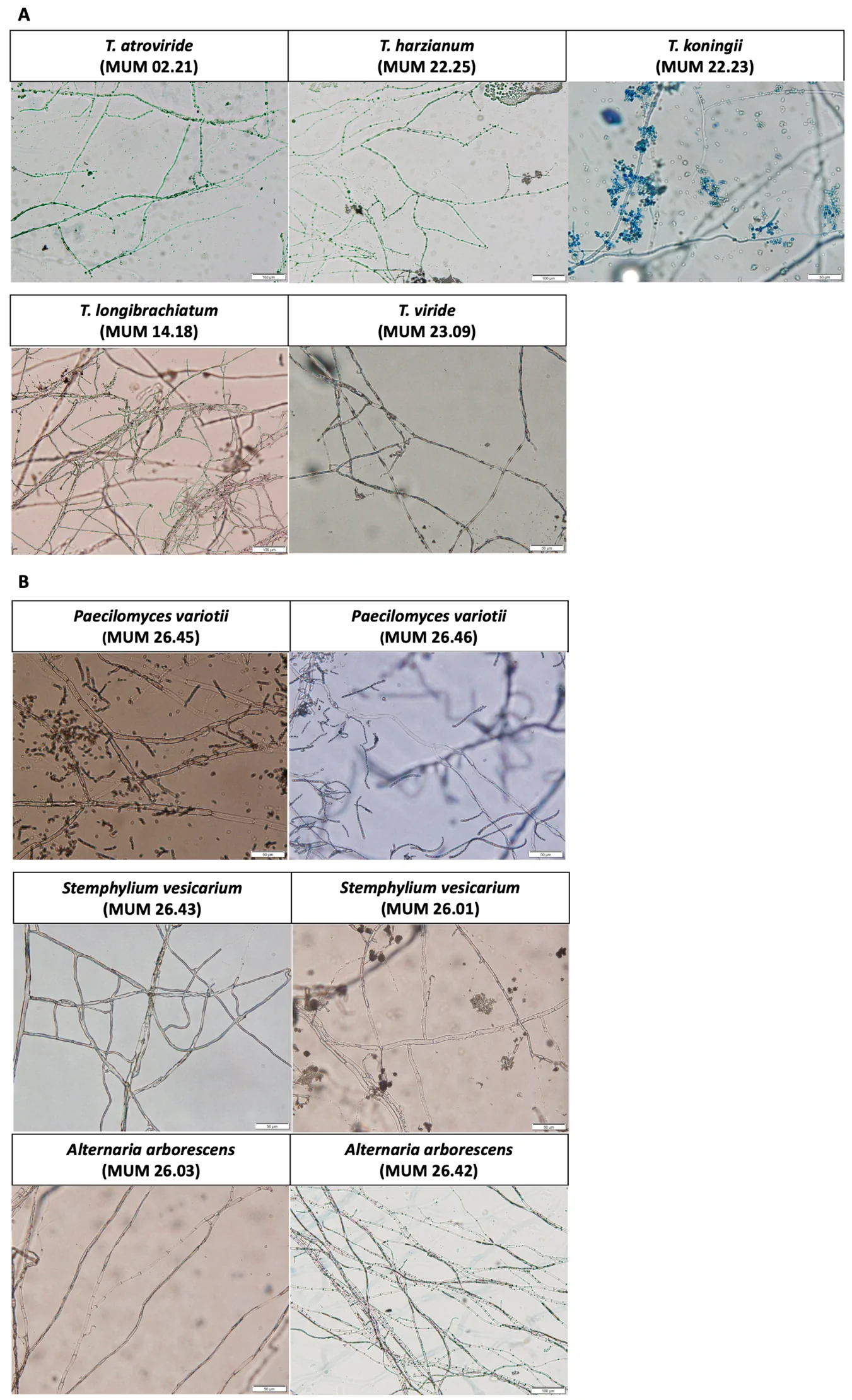
Representative microscopic images showing the hyphal morphology of (**A**) selected *Trichoderma* strains, *T. atroviride* (MUM 02.21), *T. harzianum* (MUM 22.25), *T. koningii* (MUM 22.23), *T. longibrachiatum* (MUM 14.18), and *T. viride* (MUM 23.09), and (**B**) target fungi, *P. variotii* (MUM 26.45 and MUM 26.46), *S. vesicarium* (MUM 26.43 and MUM 26.01), and *A. arborescens* (MUM 26.03 and MUM 26.42). Scale bar correspond to 50 um.

Microscopic examination of the interactions between *Trichoderma* strains and the target pathogenic fungi revealed several types of hyphal interactions, including adhesion, coiling, penetration, hyphal deformation, and lysis (Figure 7 and Figure A1).

Interactions between *T. atroviride* (MUM 02.21) and both *A. arborescens* strains revealed the presence of hyphal lysis (Figure 7A). *T. harzianum* (MUM 22.25) showed hyphal adhesion to *P. variotii* (MUM 26.45), while coiling, penetration, and hyphal lysis were observed in the interaction with MUM 26.46 (Figure 7B). Penetration of *P. variotii* (MUM 26.46) hyphae was also observed in the presence of *T. koningii* (MUM 22.23) (Figure 7C). *T. longibrachiatum* (MUM 14.18) was observed coiling around the hyphae of both *S. vesicarium* isolates. In contrast, its interaction with *A. arborescens* (MUM 26.03) involved adhesion, penetration, and deformation of the target fungal hyphae (Figure 7D). *T. viride* (MUM 23.09) exhibited coiling in interactions with *P. variotii* (MUM 26.45) and *S. vesicarium* (MUM 26.43), while penetration and hyphal lysis were observed against *P. variotii* (MUM 26.46). Adhesion, penetration, and hyphal lysis were also observed in its interaction with *A. arborescens* (MUM 26.42) (Figure 7E).

## 4. Discussion

The evaluation of the sensitivity of fungal pathogens to traditional fungicides is a crucial step in understanding and managing disease control strategies. Thus, the inhibitory activity of six commercial fungicides formulations was evaluated at the recommended field concentrations (Figure 1). Among them, Cyprodinil + Fludioxonil showed the highest overall inhibitory activity. The high efficacy of this formulation may be associated with the combination of two active ingredients with distinct modes of action. Cyprodinil is an anilino-pyrimidine fungicide belonging to FRAC group 9, whose activity involves interference with methionine biosynthesis, whereas Fludioxonil is a phenylpyrrole fungicide belonging to FRAC group 12 and acts through interference with fungal osmotic signal transduction [28]. The high sensitivity observed in the present study is consistent with previous reports for *Alternaria* spp. Wang et al. [33] found that *A. alternata* isolates obtained from blueberry were sensitive to Fludioxonil and Cyprodinil. Furthermore, Wang et al. reported high sensitivity of *A. arborescens* isolates from mandarin fruit to fludioxonil, supporting the strong inhibitory response observed for *A. arborescens* in the present study [33]. *T. trachyspermus* showed the highest susceptibility under the tested conditions, indicating that it is less prone to resistance development and more effectively controlled by these treatments. Moreover, this study highlights varying levels of fungicide susceptibility among different fungal strains. *P. variotii* strains exhibited the lowest sensitivity to combinations such as Cyflufenamid + Difenoconazole, Difenoconazole + Mandipropamid, and Metalaxyl-M + Cymoxanil, suggesting a higher degree of resistance. Similarly, certain *S. vesicarium* strains showed reduced susceptibility, with less sensitive to Cyflufenamid + Difenoconazole and to Metalaxyl-M + Cymoxanil. Among *Alternaria* strains, demonstrated decreased sensitivity to Metalaxyl-M + Cymoxanil, and some also to Difenoconazole + Mandipropamid. Nevertheless, the variable response observed among fungal isolates highlights the limitations associated with relying exclusively on chemical control.

In this study, the growth dynamics of all tested fungal pathogens (Figure 2) were first evaluated to determine the optimal incubation period for subsequent dual assays. Overall, differences in growth kinetics were observed among species and isolates evaluated in this study. *P. variotii* showed a particularly rapid growth phenotype and, in contrast, *S. vesicarium* and *T. trachyspermus* displayed the lowest growth rates under the tested conditions. Differences were also evident between isolates belonging to the same species, particularly for *S. vesicarium*. Together, these findings indicate that fungal growth characteristics may vary not only among species but also among strains. Considerable variation in growth kinetics was also observed among the *Trichoderma* strains (Figure 3), with *T. longibrachiatum* showing the highest growth rate. Rapid mycelial growth may represent an advantageous trait for *Trichoderma* biocontrol, as rapid substrate colonization can enhance competition for space and nutrients, as reported by Sawant et al. [34].

The dual-culture assay indicated that all *Trichoderma* tested exhibited antagonistic activity against the fruit-associated pathogens with all values above 30%, although the magnitude of growth inhibition varied considerably among antagonist-target combinations (Figure 4). This variability in antagonistic activity is consistent with previous studies. For instance, Bachhav et al. reported significant differences in the inhibition of *S. vesicarium* by different *Trichoderma* spp. [33]. Nevertheless, the wide range of inhibition observed in the present study indicates that antagonistic efficacy was not determined solely by the identity of *Trichoderma*, but rather by the specific interaction established between the antagonist and the target fungus. Similar target-dependent responses have been reported by Yassin et al. [31] who observed that the inhibition levels of *T. viride* and *T. harzianum* varied depending on the fungal pathogen tested.

Interestingly, antagonistic efficacy was not directly associated with intrinsic mycelial growth rate (Figure 3). Although *T. longibrachiatum* displayed the highest growth rate among the *Trichoderma* strains evaluated, it did not exhibit the greatest overall inhibition in dual culture (Figure 4). Rapid growth may provide a competitive advantage through faster colonization and competition for space and nutrients; however, the present findings indicate that this characteristic alone cannot account for the antagonistic performance observed. Antagonism by *Trichoderma* is known to involve the combined contribution of multiple mechanisms, including competition for space and nutrients, antibiosis mediated by extracellular metabolites, and direct mycoparasitic interactions [35]. These mechanisms were further investigated in the present study through culture-filtrate of *Trichoderma* (Figure 5) and microscopic interaction observation (Figure 7).

Since chemical fungicides and *Trichoderma*-based biological control were evaluated against the same group of fruit-associated fungal pathogens, comparison of their inhibition profiles provides insight into the response of these pathogens to distinct control approaches. The fungicide assays revealed marked differences in efficacy depending on both the formulation and the fungal isolate, with some isolates showing high susceptibility to certain fungicides but considerably lower susceptibility to others. This variability, which was also observed between isolates belonging to the same fungal species, represents an important limitation of relying exclusively on chemical control, since the effectiveness of a given treatment may depend strongly on the target isolate. Variability was also observed in the dual-culture assays, as the magnitude of growth inhibition depended on the specific *Trichoderma*–pathogen combination. However, despite this variation, antagonistic activity was detected in every combination tested, with inhibition values consistently above 30% (Figure 4). Thus, although the efficacy of *Trichoderma* was also strain- and pathogen-dependent, the consistent antagonistic activity observed across all tested fungal isolates supports the potential of these strains as biological control agents within integrated disease management strategies, particularly considering the variable efficacy observed among the tested chemical fungicides.

The cell- and spore-free culture supernatants of the *Trichoderma* strains retained antifungal activity against the tested pathogenic fungi, indicating that at least part of the antagonistic effect is mediated by extracellular compounds released during *Trichoderma* growth (Figure 5). Importantly, the antifungal activity observed was not directly associated with the acidity of the culture supernatants (Table 4) and is compatible with the activity of extracellular antifungal compounds. Among the strains evaluated, the culture supernatants of *T. atroviride* and *T. longibrachiatum* showed the highest overall antifungal activity (Figure 5). The production of antifungal extracellular compounds by these *Trichoderma* has also been reported previously. Imran et al. [36] demonstrated that culture supernatants of *T. atroviride* and *T. longibrachiatum* significantly inhibited the mycelial growth of *Alternaria solani*, although the magnitude of inhibition differed among the two species.

Interestingly, the relative performance of the *Trichoderma* strains differed between the dual-culture (Figure 4) results. *T. viride* exhibited the highest overall antagonistic activity in dual culture, whereas its cell- and spore-free culture filtrate showed comparatively lower inhibitory activity (Figure 5). Conversely, *T. longibrachiatum* was highly effective in dual culture (Figure 4) and it produced one of the most potent culture supernatants (Figure 5). These findings indicate that strong antagonism in dual culture does not necessarily correspond to strong activity of the corresponding cell-free filtrate and suggest that different *Trichoderma* strains may rely to different extents on distinct antagonistic mechanisms. The inhibitory activity of the culture supernatants was also strongly dependent on the target fungus. The filtrate of *T. atroviride* was particularly effective against *P. variotii* isolates but considerably less active against the remaining fungi. Differences in metabolite composition among *Trichoderma* strains, together with differences in the intrinsic susceptibility of the target fungi, may contribute to these target-dependent responses.

Heat treatment (Figure 6) provided an additional information regarding the possible nature of the compounds involved in the antifungal activity [37]. Autoclaving *T. longibrachiatum* (Figure 6A) and *T. atroviride* (Figure 6B) culture supernatants reduced its antifungal activity against all fungal isolates tested, indicating that a proportion of the inhibitory effect was associated with heat-labile components. *Trichoderma* spp. are known to produce several extracellular enzymes associated with fungal antagonism, including chitinases, β-glucanases, and proteases, which can contribute to the degradation of fungal cell walls [38]. Notably, autoclaving reduced but did not completely eliminate antifungal activity, suggesting that the overall activity of the culture supernatants may result from a mixture of compounds with different thermal stabilities and other mechanisms of action.

Microscopic examination (Figure 7) revealed several types of direct hyphal interactions between *Trichoderma* strains and the target fungi, including adhesion, coiling, penetration, hyphal deformation, and lysis. These morphological features are commonly associated with the mycoparasitic behavior of *Trichoderma* spp. and are consistent with recent microscopic studies describing similar interaction patterns during contact with fungal hosts [34,39]. Importantly, the type of interaction observed was not identical across antagonist–target combinations, suggesting that mycoparasitic behavior may depend on the specific fungal isolate encountered. These microscopic observations (Figure 7) also complement the antagonistic activity detected in the dual culture (Figure 4) and antifungal activity of culture supernatants (Figure 5). Although all *Trichoderma* strains inhibited the growth of the tested fungi in dual-culture assays, this approach alone does not distinguish the mechanisms responsible for growth suppression. The direct hyphal interactions observed by microscopy therefore provide morphological evidence that mycoparasitism may contribute to the antagonistic activity of the selected strains.

Beyond their biological and mechanistic relevance, the results obtained in this study have direct implications for the agricultural and agro-industrial sectors, where regulatory pressure and market demand for sustainable solutions are rapidly reshaping plant protection strategies. Demonstrating that different *Trichoderma* spp. can effectively suppress economically significant fungi—including *S. vesicarium*, *A. arborescens*, *P. variotii*, and *T. trachyspermus*—highlights their potential as viable alternatives to conventional fungicides.

From a commercial perspective, these findings reinforce the opportunity to develop new biocontrol formulations that are more targeted and aligned with industry needs. The clear differences observed among *Trichoderma* spp. and strains pave the way for selecting high-performance isolates suitable for real-world agricultural conditions, enabling the creation of products with greater consistency and predictability. Moreover, understanding the regional distribution of pathogen genotypes is essential for designing biocontrol products that remain effective across diverse production environments, particularly in areas where certain strains exhibit reduced fungicide sensitivity or heightened aggressiveness. Integrating *Trichoderma* into pre- and postharvest protection programs may therefore reduce losses, improve final product quality, and enhance competitiveness across fruit production chains.

An additional consideration regarding the agricultural application of *Trichoderma* spp. is their capacity to produce extracellular hydrolytic enzymes, including cellulases, which enable the degradation and utilization of lignocellulosic substrates [40]. This characteristic contributes to their ecological versatility and has supported their use in the decomposition of plant residues [41]. Therefore, cellulolytic capacity alone cannot be considered indicative of phytotoxicity, and the outcome of the interaction is likely to depend on the fungal strain, host species, plant tissue and application conditions [42]. As the present study was restricted to in vitro antagonistic assays, potential effects of the selected isolates on living fruit-tree tissues were not assessed. Consequently, before their implementation under orchard conditions, further in vivo studies should evaluate their compatibility with the host plant, including possible phytotoxic or tissue-degrading effects on foliage, fruits and woody tissues, together with the optimization of inoculum concentration and application strategy.

## 5. Conclusions

This work highlights that *Trichoderma* spp. exhibit significant biocontrol potential against key fungi pathogens affecting fruit production, primarily through extracellular antifungal compounds and direct mycoparasitic interactions, offering a sustainable alternative to chemical fungicides.

Among the strains evaluated, *T. viride* and *T. harzianum* showed particularly strong antagonistic activity in dual-culture assays. Microscopic observations further supported the occurrence of direct antagonistic interactions, revealing morphological alterations in the pathogen hyphae associated with their interaction with *Trichoderma.* Regarding extracellular antifungal activity, *T. atroviride* and *T. longibrachiatum* produced the most active culture supernatants, with *T. atroviride* showing pronounced inhibitory activity against *P. variotii* and *T. longibrachiatum* exhibiting strong activity against a broader range of the tested fungal isolates. Autoclaving markedly reduced the antifungal activity of these culture supernatants without completely eliminating it, suggesting the contribution of extracellular compounds with different thermal stabilities. Nevertheless, as the present study was restricted to in vitro assays, further in vivo studies are required to confirm the compatibility of the most promising *Trichoderma* strains with fruit-tree tissues and to exclude potential phytotoxic or tissue-degrading effects associated with their hydrolytic activity before application under orchard conditions.

Additionally, this study highlights the importance of understanding fungal growth dynamics, and the complex, strain-dependent mechanisms underlying *Trichoderma*’s antagonistic activity, which collectively support the development of integrated, environmentally friendly disease management strategies.

## Figures and Tables

**Table 1:** Sources, species, and identification codes of fruit-associated fungal pathogens.

Sources	Species	Codes	Geographical Origin
Juice fruit matrices	*Talaromyces trachyspermus*	MUM 26.13	Portugal
	*Paecilomyces variotii*	MUM 26.45	Portugal
	*Paecilomyces variotii*	MUM 26.46	Portugal
Pears with BSP	*Stemphylium vesicarium*	MUM 26.43	Portugal
	*Stemphylium vesicarium*	MUM 26.01	Portugal
	*Alternaria arborescens*	MUM 26.03	Portugal
	*Alternaria arborescens*	MUM 26.42	Portugal

**Table 2:** *Trichoderma* strains used during this study.

*Trichoderma* spp.	Codes	Substrate/Host	Geographic Origin
*T. alni*	MUM 22.22	*Fucus* sp.	Aveiro, Portugal
*T. atroviride*	MUM 02.21	Spring water	Portugal
*T. harzianum*	MUM 22.25	Algae	Portugal
*T. koningii*	MUM 22.23	Gracilaria gracilis	Aveiro, Portugal
*T. koningiopsis*	MUM 14.19	Maize	Plateau State, Nigeria
*T. longibrachiatum*	MUM 14.18	Guinea corn	Plateau State, Nigeria
*T. saturnisporum*	MUM 22.24	Sponge	Aveiro, Portugal
*T. tomentosum*	MUM 22.21	Sponge	Aveiro, Portugal
*T. viride*	MUM 23.09	Animal food	*
*T. viride*	MUM 9754	*Fagus sylavatica*, dead trunlk	Germany

* No information available.

**Table 4:** pH values of culture supernatants obtained from *Trichoderma* grown in PDB at 25 °C.

*Trichoderma* spp.	Codes	pH
*T. atroviride*	MUM 02.21	4.63 ± 0.21
*T. harzianum*	MUM 22.25	5.92 ± 0.45
*T. koningii*	MUM 22.23	4.02 ± 0.49
*T. longibrachiatum*	MUM 14.18	5.63 ± 0.36
*T. viride*	MUM 23.09	4.76 ± 0.16

## Data Availability

The raw data supporting the conclusions of this article will be made available by the authors on request.

## Abbreviations

The following abbreviations are used in this manuscript:

ANOVA	Analysis of Variance
BCA	Biological Control Agent
BSP	Brown Spot of Pear
CEB	Centre of Biological Engineering
FCT	Portuguese Foundation for Science and Technology
INIAV	National Institute for Agrarian and Veterinary Research
ITS	Internal Transcribed Spacer
LABBELS	Associate Laboratory in Biotechnology, Bioengineering and Microelectromechanical Systems
MALDI-TOF MS	Matrix-assisted Laser Desorption/Ionization Time-of-flight Mass Spectrometry
MGI	Mycelial Growth Inhibition
MUM	Micoteca University of Minho
PDA	Potato Dextrose Agar
PDB	Potato Dextrose Broth
PIRG	Percentage Inhibition of Radial Growth
UM	University of Minho

## Appendix A

The hyphal morphology of the selected *Trichoderma* strains and target fungal isolates was examined by optical microscopy, and representative images are presented in Figure A1.
